# Evaluation of a decentralized investigational drug service pharmacist in a cancer clinical trial infusion unit

**DOI:** 10.1177/10781552231207854

**Published:** 2023-10-17

**Authors:** Christina Baroody, Melissa Sandler, Christine Hong, Yazan F Madanat, Stefanie Conley

**Affiliations:** 12334 University of Texas Southwestern Medical Center, Dallas, TX, USA

**Keywords:** Decentralization, oncology infusion unit, investigational drug service, clinical pharmacist, clinical trials

## Abstract

**Introduction:**

Investigational drug service (IDS) oversees and manages use of investigational products. There is limited data on utility of pharmacy services in clinical trial conduct and outcomes, specifically on the value of a decentralized IDS pharmacist.

**Methods:**

This is a quasi-experimental study conducted in an oncology clinical trial infusion unit. A retrospective chart review was done to reflect current practice from January through June 2022. A decentralized IDS pharmacist was piloted in December 2022. Data collected included number and types of consults, personnel requesting the consult, and intervention performed. A satisfaction questionnaire was conducted after the pilot program.

**Results:**

A total of 16.3% (173 of 1062 patient visits) of pharmacy consults were completed in the centralized IDS pharmacy model, while 44.5% (81 of 182 patient visits) of pharmacy consults were completed during the decentralized IDS pharmacist pilot, *p* < .001. Decentralized IDS pharmacist completed 77% (62/81) of the consults during the pilot period. Most common types of consults were toxicity management (20%), electronic medical record issues (17%), and tubing and drug administration issues (16%). More than 80% of respondents to the satisfaction questionnaire responded that implementation of a decentralized IDS pharmacist is acceptable, appropriate, and feasible.

**Conclusion:**

This pilot study demonstrated that a decentralized IDS pharmacist in an oncology clinical trial infusion unit improved accessibility to an IDS pharmacist, increased pharmacy consults relevant to patient care and optimized centralized pharmacists medication distribution workflow. Further studies are needed to evaluate patient benefits from implementing decentralized IDS pharmacist in direct patient care activities.

## Introduction

The American Society of Clinical Oncology (ASCO) emphasizes the importance of multidisciplinary teams for the success of clinical cancer research. These multidisciplinary teams optimize operational activities, patient recruitment, as well as cancer treatment, which set the standard for an exemplary clinical trial site.^1^ The investigational drug service (IDS) is a research pharmacy that oversees and manages the use of investigational products (IP). As part of the multidisciplinary team, IDS pharmacists contribute unique insights from administrative, operational, clinical, and patient safety perspectives.

Some of the obstacles encountered in clinical trials are distinctive to research and unlikely to occur with commercially available products. Protocol complexity, lack of safe practice standardization, and an unknown toxicity profile for many IP contribute to these risks.^2^ In order to mitigate these consequences, IDS pharmacists focus on the following tasks, but their duties not limited to: maintain drug accountability, manage IP preparation, monitor research participants, educate healthcare providers, and build IP into the electronic health record for computerized provider order entry. IDS pharmacists have comprehensive knowledge of the clinical trial protocol, eligibility criteria, drug interactions, and toxicities associated with IP. IDS best practices have evolved throughout the years as a result of an increasing number of clinical trials and complexity of clinical trial designs.^3,4^ With progressively complicated drug regimens, pharmacists’ roles in direct patient care are growing, creating a need for pharmacy departments to expand their scope of services.

Despite the various roles that IDS pharmacists serve in clinical research, there is limited data on the impact of pharmacy services in clinical trial conduct and outcomes. There have been published studies to demonstrate improved patient safety by increasing pharmacist involvement in prescription review, education, monitoring, and reviewing for drug interactions. One retrospective study in Korea found that IDS pharmacist interventions significantly improved medication safety by reducing prescribing errors in cancer research participants.^5^ A cross-sectional survey of 21 teaching hospitals in France found that only a quarter of adult patients had complete understanding of the investigational drugs they were taking. The article suggested that clinical trial sponsors should encourage initiatives aimed at educating participants, which can be fulfilled by the IDS pharmacists.^6^ A prospective study published in 2021 evaluated the use of a hospital pharmacist in reviewing the medication list of research participants for drug interactions at clinical trial inclusion. The hospital pharmacist categorized patients as either low or high risk for drug interactions. To confirm the risk assessment, the study conducted a pharmacokinetic analysis that demonstrated a higher and potentially toxic serum concentration of study drug in patients who the pharmacist deemed as high risk.^7^ Lastly, a national survey of 68 National Cancer Institute-designated cancer centers highlighted that the majority of services for clinical trials were viewed as important for pharmacists to perform; however, less than half of these services were performed by a pharmacist more than 50% of the time.^8^ Overall, this body of literature demonstrates a potential to optimize the care for research participants through increased pharmacist involvement in a variety of direct patient care activities.

At University of Texas Southwestern Medical Center, Harold C. Simmons Comprehensive Cancer Center, the research participants receive IP in the clinical trial infusion unit. IDS pharmacy clinical and operational supports are centralized in the infusion pharmacy. The infusion clinical pharmacists are responsible for verifying the research orders and work in collaboration with IDS pharmacists. IDS pharmacists are centralized and function remotely from the patient care area for clinical consultation. Primary communication tools for pharmacy consults include, but are not limited to, phone, email, or instant messaging within electronic health records. IDS pharmacists screen the patients for eligibility, perform IP accountability and dispensing, participate in research committees, build IP in the electronic health record, and facilitate the site audits. Investigational drug inquiries by the nurses or other healthcare professionals are directed to the centralized IDS pharmacy due to lack of IDS pharmacist presence in the infusion unit. The limitation of this centralized model may limit the clinical consultation provided by an IDS pharmacist. Therefore, this pilot study aims to define the role of a decentralized IDS pharmacist, to evaluate the impact of a decentralized IDS pharmacist in the infusion unit on pharmacist interventions, and to assess the research team's satisfaction with the implementation of a decentralized IDS pharmacist.

## Methods

This is a quasi-experimental pilot study conducted at the University of Texas Southwestern Medical Center Harold C. Simmons Comprehensive Cancer Center in Dallas, Texas. To facilitate optimal patient care, our institution piloted a decentralized IDS pharmacist to respond in person or closer to patient care infusion unit to consults and drug information questions from the research team. The decentralized IDS pharmacist also proactively identified opportunities to intervene and provide education.

Retrospective data before the implementation of a decentralized IDS pharmacist was collected through a medical chart review of research patients treated January through June 2022 to reflect the current practice. The number and type of pharmacy consults, the personnel consulting pharmacy, and the intervention made by the pharmacist was collected. In December 2022, the decentralized IDS pharmacist worked in the clinical trial infusion unit for a whole month. The decentralized IDS pharmacist collected the same data as above, prospectively. At the end of the pilot program, a satisfaction questionnaire was sent via email to the research coordinators, research managers, nurses, and pharmacists. The satisfaction questionnaire was derived from a validated series of questions used to evaluate the opinion of the respondents on the acceptance, appropriateness, and feasibility of the implemented decentralized IDS pharmacist (Table 1).^9^

**Table 1.** Satisfaction questionnaire.

Category	Question
Acceptability	1. IDS Pharmacist in the Clinical Trial Infusion Unit meets my approval 2. Having an IDS Pharmacist in the clinical trial unit is appealing to me 3. I like having an IDS Pharmacist in the clinical trial infusion unit
Appropriateness	4. IDS Pharmacist in the Clinical Trial Infusion Unit seems fitting 5. IDS Pharmacist in the Clinical Trial Infusion Unit seems suitable 6. IDS Pharmacist in the Clinical Trial Infusion Unit seems applicable
Feasibility	7. IDS Pharmacist in the Clinical Trial Infusion Unit seems implementable 8. IDS Pharmacist in the Clinical Trial Infusion Unit seems possible 9. IDS Pharmacist in the Clinical Trial Infusion Unit seems doable

Responses for each item listed above were rated on a 1 to 5 scale: 1 = Completely Disagree, 2 = Disagree, 3 = Neither Agree nor Disagree, 4 = Agree, 5 = Completely Agree.

The primary endpoints were the number and type of consults documented and the intervention recommended by the IDS pharmacist. The secondary endpoint was the satisfaction of the research team regarding the decentralized IDS pharmacist in the clinical trial infusion unit assessed using the validated questionnaire.

Statistical analysis of the primary endpoint was calculated using the Chi-squared test. Descriptive statistics were used to assess the findings. Percent utilization was calculated by dividing the total number of documented pharmacist interventions by the number of patient appointments.

## Results

From January to June 2022, there were 1062 appointments scheduled for research patients; 173 pharmacy consults were documented in their medical charts which represents about 16.3% of consults). During the 1-month pilot period, there were 182 appointments scheduled for research patients and 81 pharmacy consults were prospectively documented which represents about 44.5% utilization (*p* < .001).

### Pre-decentralized IDS pharmacist

**Figure 1.**
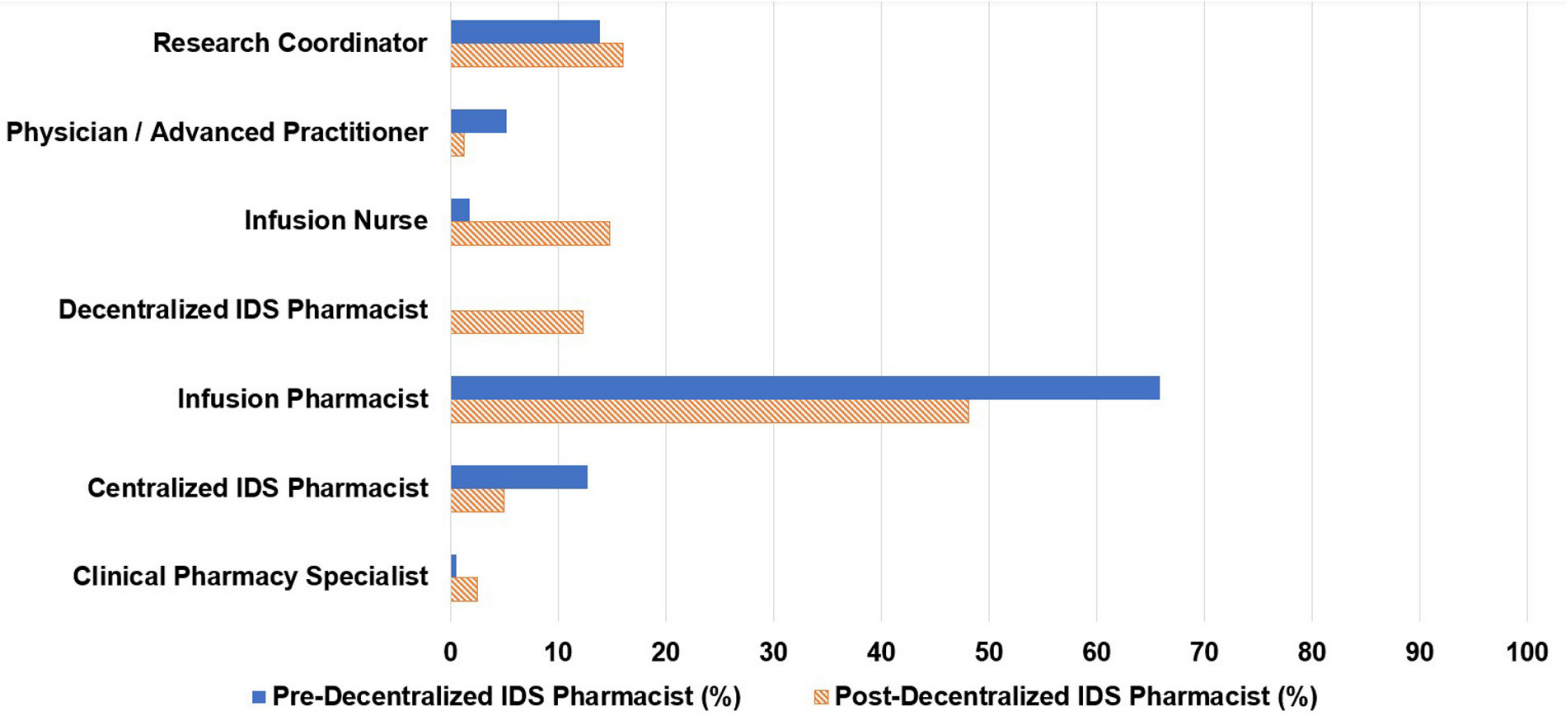
Personnel requesting consult.

**Figure 2.**
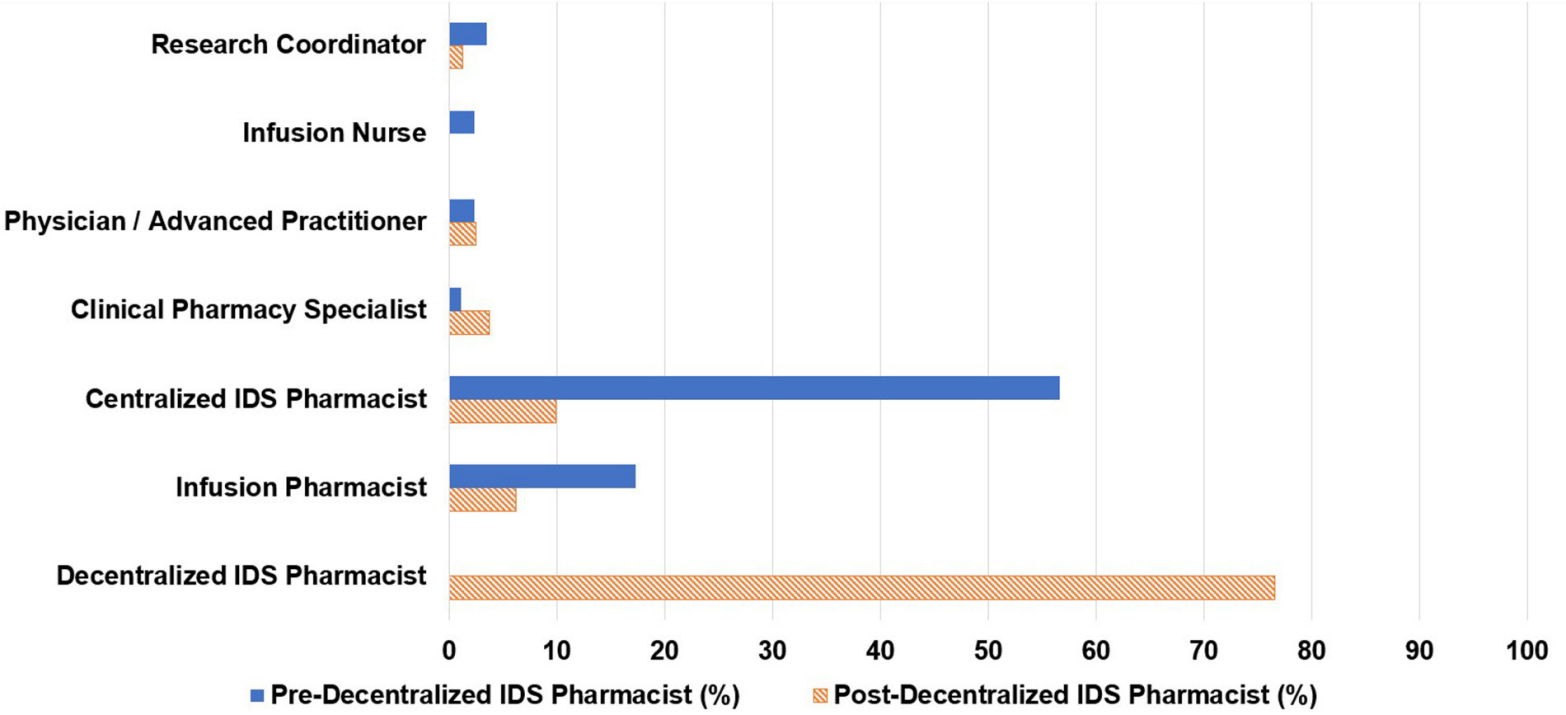
Personnel responding to consult.

**Figure 3.**
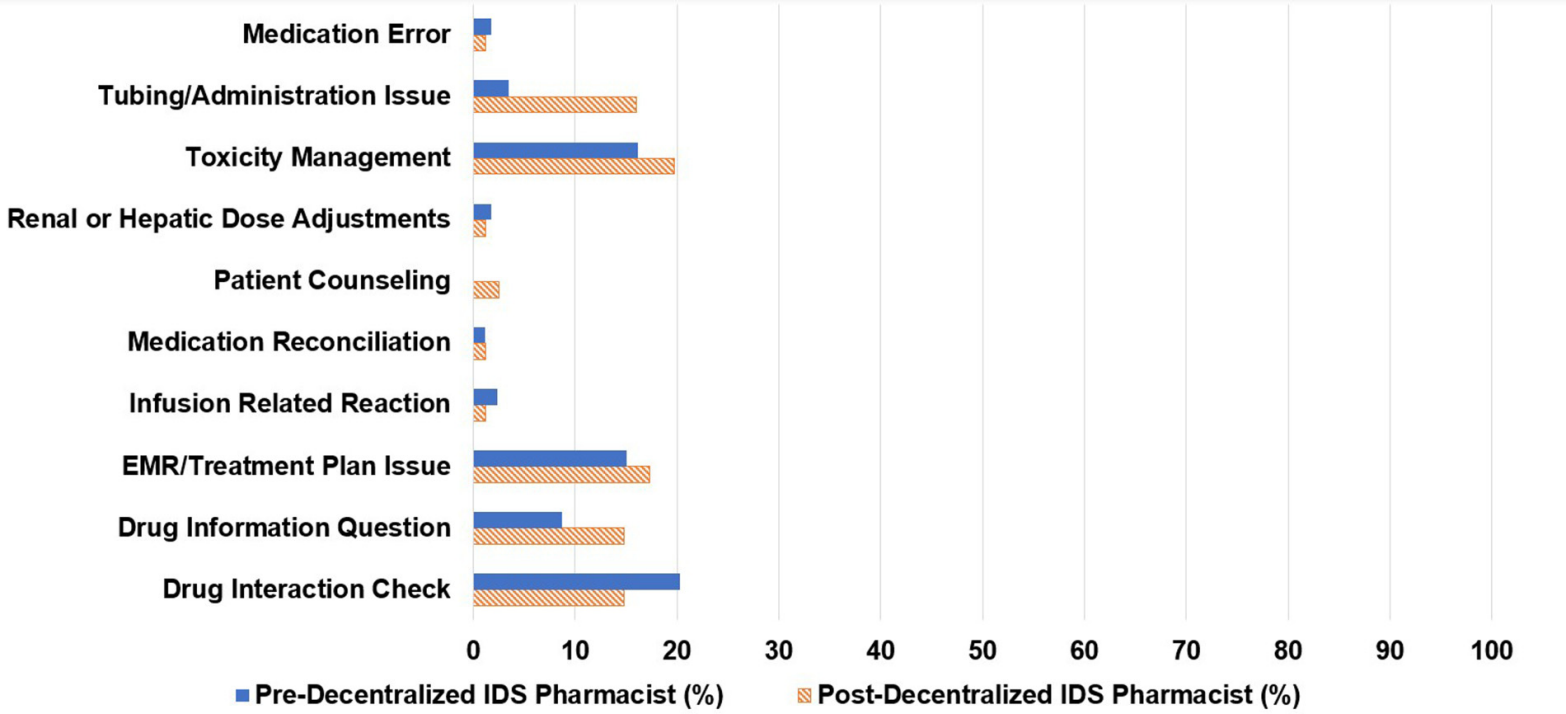
Types of pharmacist consults.

Of the 173 consults, 66% were initiated by infusion pharmacists, 14% by research coordinators, and 13% by centralized IDS pharmacists (Figure 1). Centralized IDS pharmacists responded to 57% of consults and infusion pharmacists responded to 17% (Figure 2). The most common types of consults were drug interaction checks (20%), toxicity management (16%), and issues in the electronic medical record (15%) (Figure 3).

### Decentralized IDS pharmacist pilot period

**Figure 4.**
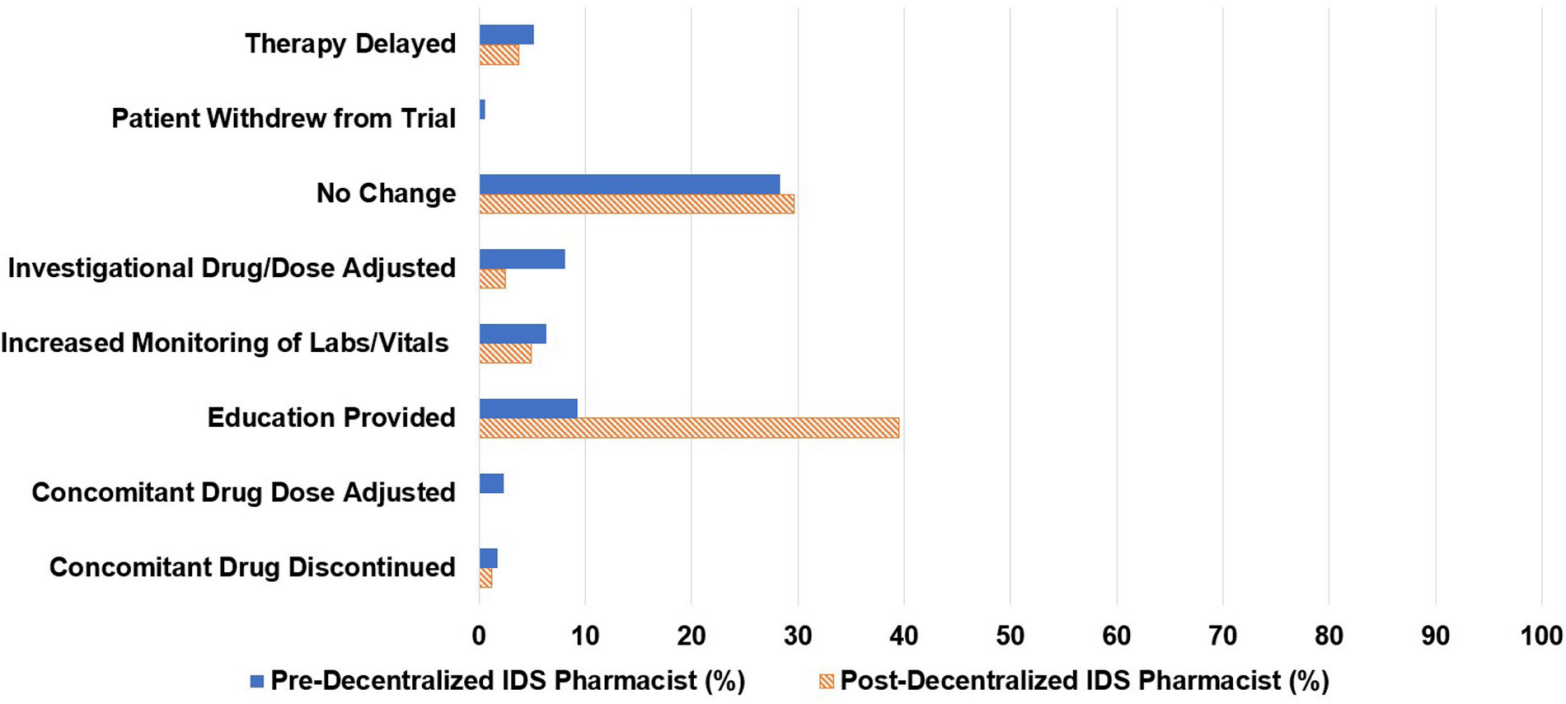
Pharmacist interventions performed.

Of the 81 pharmacy consults, 48% were initiated by infusion pharmacists, 16% by research coordinators, 15% by infusion nurses, and 5% by centralized IDS pharmacists. The decentralized IDS pharmacist proactively initiated 12% of the consults (Figure 1). The decentralized IDS pharmacist responded to 77% of these consults (Figure 2). The most common types of consults received were toxicity management (20%), issues in the electronic medical record (17%), tubing and drug administration issues (16%), drug information questions (15%), and drug interaction checks (15%) (Figure 3). The decentralized IDS pharmacist provided 29 education sessions in 1 month compared to 16 education sessions in the 6-month retrospective period (Figure 4).

### Satisfaction questionnaire

**Figure 5.**
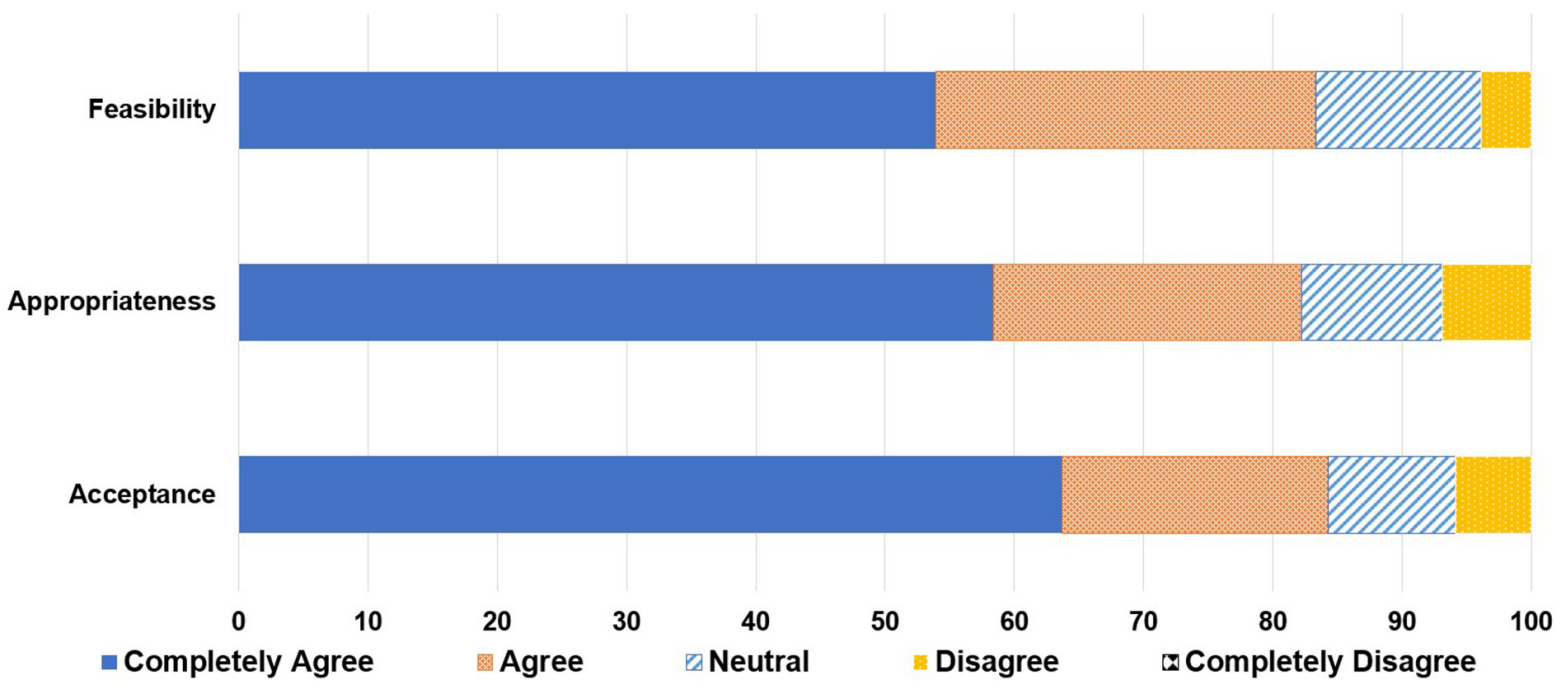
Satisfaction questionnaire results.

The questionnaire was sent to 62 participants and there were 34 responses (65% response rate). More than 80% of respondents agreed that the implementation of a decentralized IDS pharmacist is acceptable, appropriate, and feasible (Figure 5).

## Discussion

Decentralization in health systems is defined as moving the decision-making closer to users of the health services. Decentralized clinical pharmacists are integrated into patient care teams to provide bedside services such as therapeutic drug monitoring, high-risk medication management, antimicrobial stewardship, and counseling. Several studies demonstrate improved patient-related outcomes and quality of care by incorporating decentralized clinical pharmacists.^10–12^ Therefore, this study aims to evaluate the extension of these decentralized clinical pharmacy services to the field of investigational drugs and research.

Our findings demonstrated an increase in opportunities for the decentralized IDS pharmacist to intervene on patient-related issues in the clinical trial infusion unit through the increase in percent utilization of pharmacy services from 16.3% to 44.5%. There was an increase in the number of consults initiated by infusion nurses which reflects expanded communication between the infusion nurses and the IDS pharmacist. Some examples of nurse-initiated inquiries that occurred during the study included drug information questions such as mechanism of action and indication, timing of investigational drug and premedication administration per clinical trial protocol, and clarifying the duration of therapy. Enhancing interdisciplinary relationships and collaboration through increased communication allow healthcare professionals to practice patient safety competency.

The addition of a decentralized IDS pharmacist in the clinical trial infusion unit increased the percentage of consults related to toxicity management, electronic medical record order clarification and modification, and drug administration questions. These concerns are readily addressed in person rather than as a centralized remote service. A decentralized IDS pharmacist in the clinical trial infusion unit promoted timely response to consults and an opportunity to intervene on issues that might have been undetected by centralized pharmacists if they were not notified. Additionally, the decentralized IDS pharmacist increased the number of education sessions by about 10 times the number of sessions per month. The educational sessions included explaining investigational drug dosing, clarifying required safety parameters, and creating training documents, which allowed for streamlined workflow and adherence to clinical trial protocols. An increase in these types of consults was expected as the decentralized IDS pharmacist served as a convenient access to expert source of information.

The volume of clinical support distribution among the pharmacy staff (centralized IDS pharmacists and infusion pharmacists) was also analyzed. There was a reduction in the centralized IDS pharmacist clinical consult support from 57% to 10% as the decentralized IDS pharmacist responded to the majority of consults (Figure 2). This allowed centralized pharmacists to re-allocate their time to focus on central responsibilities such as order verification and timely dispenses.^13^

Responses to the satisfaction questionnaire indicated an agreeable attitude by the research team toward the implementation of a decentralized IDS pharmacist in terms of acceptability, appropriateness, and feasibility. This implies that there will be well-received efforts for interdisciplinary collaboration and providing well-rounded care to research patients as outlined in the ASCO standards for exemplary clinical trial sites.^1^

The findings of this study align with efforts to advance pharmacy practice by expanding the scopes of services provided by IDS pharmacists and developing a new position for an IDS pharmacist that is directly involved in patient care activities, which has not yet been documented in published literature.^14^ Interdisciplinary collaboration and the opportunity for increased pharmacy interventions promotes medication safety and establishing successful sites for clinical trial conduct.

This is the first study to evaluate the decentralization of IDS pharmacy services in a clinical trial infusion unit. It showed increased accessibility to the decentralized IDS pharmacist as well as satisfaction from the study team. Our results are limited by the duration of the 1-month pilot period and descriptive statistics without power calculations. Significantly, given the retrospective nature of the pre-decentralized IDS pharmacist data, this study was limited due to potential lower rates of documentation in the research patients’ electronic medical records on pharmacy interventions during the pre-decentralized IDS pharmacist period. In contrast, during the post-decentralized IDS pharmacist period, the pharmacist prospectively captured all pharmacist interventions performed; this may have led to performance bias. Finally, the scope of the project was limited to specific, achievable interventions in order to prevent confusion associated with significant workflow changes and to allow time for detailed documentation for research purposes. A decentralized IDS pharmacist would be responsible for more education and interventions than included in this pilot.

Moving forward, this study supports the benefit of a decentralized IDS pharmacist that is directly involved in patient care activities such as medication reconciliation, education, adverse event monitoring, drug information provision, educational in-services, and electronic medical record troubleshooting.

## Conclusion

In conclusion, a decentralized IDS pharmacist increased the percent pharmacy consult for IDS pharmacy, increased accessibility to IDS pharmacy services, and decreased centralized pharmacists’ to address clinical consult and focus on central operational activities for patient care. Further studies are required to fully assess the impact this role would have on patient-related outcomes as well as potential financial implications associated with adding a new position for a decentralized IDS pharmacist.
